# The efficacy of medial meniscal posterior Root tear Repair with or without high tibial osteotomy: a systematic review

**DOI:** 10.1186/s12891-023-06520-9

**Published:** 2023-06-06

**Authors:** Hangle Wang, Qian Man, Yitian Gao, Lingyi Xu, Jingwei Zhang, Yong Ma, Qingyang Meng

**Affiliations:** 1 Department of Sports Medicine, Beijing Key Laboratory of Sports Injuries, Peking University Third Hospital, Institute of Sports Medicine of Peking University, 49 North Garden Road, Haidian District, Beijing, 100191 People’s Republic of China; 2 Peking University Health Science Center, 38 Xueyuan Road, Haidian District, Beijing, 100191 People’s Republic of China; 3 Peking Unversity First Hospital, 8 Xishiku Street, Xicheng District, Beijing, 100034 People’s Republic of China; 4 Peking University People’s Hospital, No.11 Xizhimen South Street, Xicheng District, Beijing, 100044 People’s Republic of China; 5 Engineering Research Center of Sports Trauma Treatment Technology and Devices, Ministry of Education, Beijing, People’s Republic of China

**Keywords:** Medial meniscal posterior root tear, High tibial osteotomy, Varus alignment, Clinical assessment, Radiologic outcome

## Abstract

**Background:**

Medial meniscal posterior root tear (MMPRTs) is a common lesion of the knee joint, and repair surgery is a well-established treatment option. However, patients with obvious varus alignment are at an increased risk for MMPRT and can suffer from a greater degree of medial meniscus extrusion, which leads to the development of osteoarthritis following repair. The efficacy of high tibial osteotomy (HTO) as a means of correcting this malformation, and its potential benefits for MMPRT repair, remains unclear.

**Purpose:**

To explore whether HTO influenced the outcome of MMPRT repair in clinical scores and radiological findings.

**Study design:**

Systematic review.

**Methods:**

According to the PRISMA (Preferred Reporting Items for Systematic Review and Meta-Analyses) guidelines, we searched PubMed, Embase, Web of Science, and the Cochrane Library databases for studies reporting the outcomes of MMPRT repair and extracted data about characteristics of patients, clinical functional scores and radiologic outcomes. One reviewer extracted the data and 2 reviewers assessed the risk of bias and performed a synthesis of the evidence. Articles were eligible if they reported the results of MMPRT repair with exact mechanical axis (registered in the International Prospective Register of Systematic Reviews, CRD42021292057).

**Results:**

Fifteen studies with 625 cases of high methodological quality were identified. Eleven studies were assigned to the MMPRT repair group (M) with 478 cases performing MMPRT repair only, and others belonged to the MMPRT repair and HTO group (M and T) performing HTO and MMPRT repair. Most of the studies had significantly improved clinical outcome scores, especially in M groups. And the radiologic outcomes showed that the osteoarthritis deteriorated in both groups with similar degree in about 2-year follow-up.

**Conclusion:**

HTO is a useful supplement in treating MMPRT patients with severe osteoarthritis and the clinical and radiological outcomes were similar with MMPRT repair alone. Which would be better for patients’ prognosis generally, performing MMPRT repair alone or a combination of HTO and MMPRT repair, was still controversial. We suggested taking K-L grade into account. Large-scale randomized control studies were called for in the future to help make better clinical decisions.

**Level of evidence:**

III

## Introduction

The meniscal root tear is an avulsion injury or radial tear located within 1 cm of the meniscus root attachment [1], with the most common being the medial meniscal posterior root tear (MMPRT) first reported by Pagnani et al. [2]. This type of tear destroys the hoop construction of the meniscus and can have a long-term, detrimental impact on joint stresses and cartilage degeneration. Therefore, functional restoration of this injury is of utmost importance.

Various treatment options for MMPRT repair have been complemented, including non-operative treatment, partial meniscectomy, and MMPRT repair [3]. Biomechanical studies have confirmed that MMPRT repair can reverse the high contact pressure of the tibiofemoral joint [4, 5]. Clinical research has consistently concluded that MMPRT repair can delay the onset of osteoarthritis and the need for knee arthroplasty, when compared to non-operative and partial meniscectomy [6, 7]. However, when there is a varus abnormality and the mechanical axis of the lower limb deviates significantly from the normal range, MMPRT repair alone may not be sufficient. In such cases, high tibial osteotomy (HTO) can be used to correct lower limb alignment and reduce the burden on the medial meniscus [8]. While the effects of combining these surgeries have been studied [9], further research is needed to definitively determine the efficacy of this approach.

Our purpose was to investigate whether high tibial osteotomy (HTO) influences the outcome of medial meniscus posterior root tear (MMPRT) repair in terms of clinical scores and radiological findings. We hypothesized that the repair of MMPRT after HTO for patients with varus alignment could lead to better results than those with normal alignment without HTO.

## Methods

### Searching strategy

The protocol of this review was registered in the International Prospective Registry of Systematic Reviews (CRD42021292057). Research of PubMed, Embase, Web of Science, and Cochrane Library databases was performed on March 30th, 2023 with the terms ((Medial meniscus[Title/Abstract]) OR (medial meniscal[Title/Abstract]) AND ([Root tear] OR [Root tears] OR [posterior root tear] OR [posterior root tears] OR [posterior horn tear] OR [posterior horn tears] OR [posterior horn root tear] OR [posterior horn root tears] OR [avulsion]) AND [repair]).

### Eligibility criteria

The inclusion criteria were as follows: (1) randomized controlled trials, observational cohort studies, and case-control studies (Level of Evidence I, II, or III); (2) Patients with medial meniscus posterior root tear (as diagnosed by a clinician or using any recognized diagnostic criteria) who underwent MMPRT repair. The exclusion criteria were as follows: (1) Patients underwent combined knee surgeries: combined osteotomy surgery, combined ligament surgery, combined cartilage restoration surgery, and combined lateral meniscal repair surgery; (2) Patients suffering from MMPRT caused by acute injuries; (3) Patients with missing information on neural or varus alignment; (4) Patients with follow-up less than 1.5 years.

Any researches that failed to meet the eligibility criteria were excluded. If data of multiple literature come from the same patient population, the article with the longest follow-ups was reserved.

### Data extraction and quality assessment

Date from included studies was extracted by two reviewers. Any controversy was resolved by further discussion with the corresponding author. The extraction included the following: (1) the basic characteristics of included studies (author, publication date, study design and duration of follow-up); (2) the details of surgeries conducted (MMPRT repair or MMPRT repair with HTO); (3) the details of radiological outcomes (IKDC, Lysholm, VAS, HSS, and Tegner activity scale, K-L grade, mechanical axis, medial joint space, meniscal extrusion, and healing status of medial meniscus). In our research, Newcastle-Ottawa Scale (NOS) was used to assess quality for cohort study.

### Statistical anaylsis

The data analysis was conducted using RevMan Manager 5.4 (Copenhagen: The Nordic Cochrane Centre, The Cochrane Collaboration, 2022). Using the same format, two reviewers independently collected data and crosschecked the results. Disagreements were discussed with the corresponding author and reached consensus in order to ensure accuracy. Odds ratio (OR) with 95% confidence interval (CI) was calculated for dichotomous while mean difference (MD) with corresponding 95% CI was calculated for continuous outcomes.

**Fig. 1:**
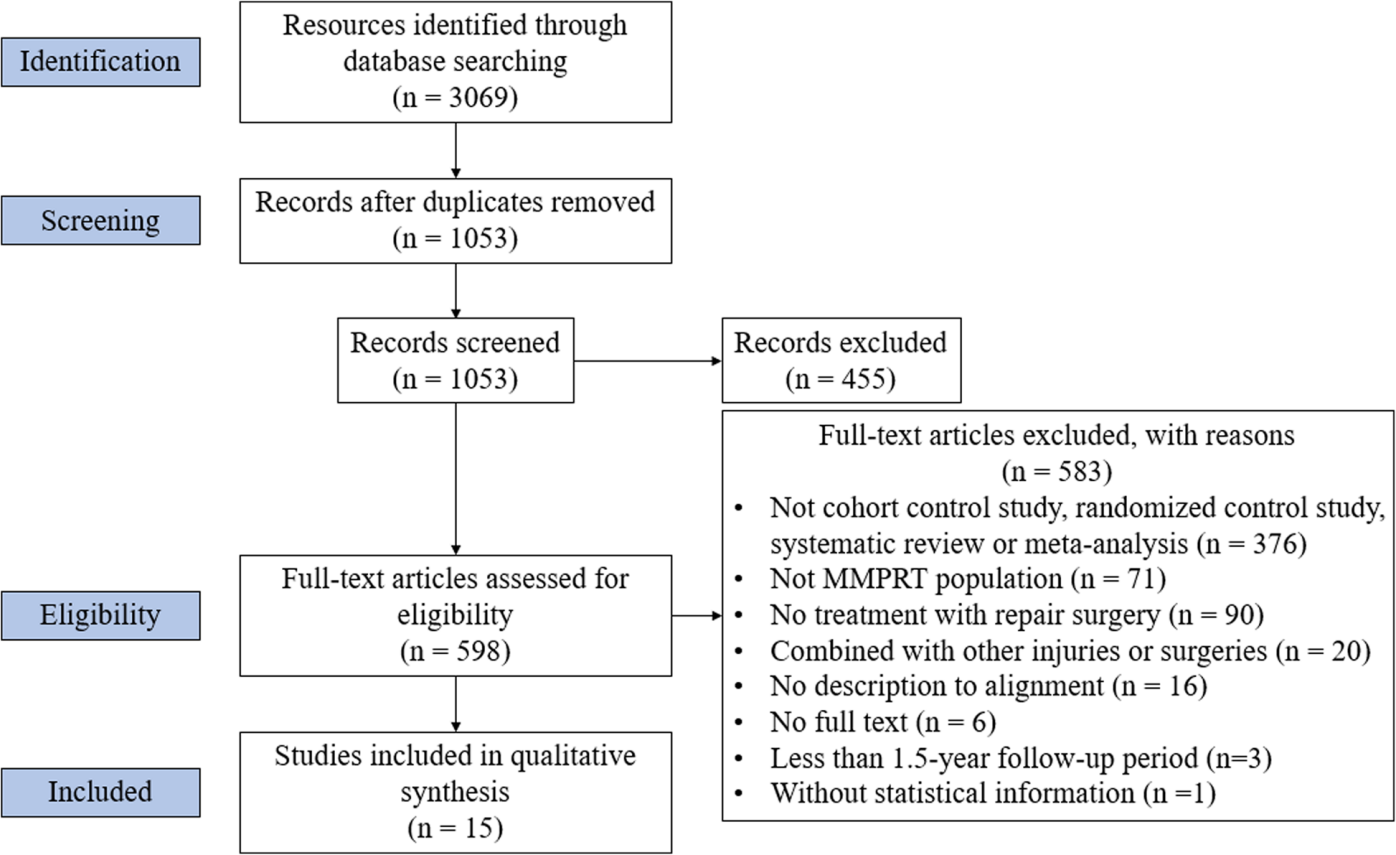
PRISMA (Preferred Reporting Items for Systematic Review and Meta-Analyses) flowchart

## Results

Finally, there were 11 papers [7, 10–19] in MMPRT repair group (M) and 4 papers [9, 20–22] in MMPRT repair + HTO group (M and T) meeting the criteria (Fig. 1). 80.0% of these studies had a level of evidence III, while 2 studies [13, 17] (13.3%) were of level IV and 1 study [9] (6.7%) was of level II. The number of knees in total was 625 (478 in M group versus 147 in M and T group). The sex ratio (male/female) was 108/474 (85/349 in M group versus 23/125 in M and T group). The mean age of all patients was 56.2 (56.8 in M group versus 56.0 in M and T group). The mean follow-up time ranged from 16.6 months [18, 23–25] to 125.9 months [7]. The quality of each article was estimated by Newcastle-Ottawa Scale (NOS), and all 15 articles were no less than 7 points. The detailed information could be seen in Table 1.

**Table 1:** Characters of the Included Studies^a^

Author(s)	Year of publication	LoE	NOS	Number of knees	Sex (M/F)	Age (mean ± SD, y)	Follow-up (mean ± SD, mo)
M group							
Kim, et al. [10]	2011	III	9	45	16/29	53.0 ± 5.6	26.4 ± 4.5
Lee, et al. [11]	2014	III	9	25	2/23	56.5 ± 6.1	25.9 ± 5.5
Chung, et al. [12]	2015	III	9	37	4/33	55.5 ± 7.1	72.0 ± 14.6
Chung, et al. [13]	2019	IV	9	47	5/42	59.8 ± 6.8	71.9 ± 19.2
Kim, et al. [14]	2019	III	8	21	2/19	55.9 ± 4.9	39.2 ± 11.4
Chung, et al. [7]	2020	III	9	37	5/32	56.8 ± 7.1	125.9 ± 21.2
Hiranaka, et al. [15]	2020	III	8	47	15/32	62.4 ± 7.9	3 (y)
Ulku, et al. [16]	2020	III	9	41	5/36	52.9 ± 3.8	44.6
Dzidzishvili, et al. [17]	2021	IV	7	44	-	45.2 ± 12.5	27.6 ± 5.0
Furumatsu, et al. [18]	2021	III	9	83	21/62	63.6 ± 8.9	16.6
Moon, et al. [19]	2021	III	9	51	10/41	55.5 ± 7.7	≥ 2 (y)
M and T group							
Ke, et al. [9]	2020	II	7	30	4/26	55.4 ± 7.2	29.0 ± 3.2
Lee, et al. [20]	2020	III	9	49	3/46	55.6 ± 6.2	27.1 ± 5.8
Lee, et al. [21]	2021	III	8	25	8/18	58.1 ± 4.2	1.9 ± 2.4(y)
Suh, et al. [22]	2021	III	8	43	8/35	55.7 ± 5.6	≥ 2 (y)

^a^LoE, Level of evidence; *NOS *Newcastle-Ottawa Scale, *M *male, *F *female, *SD *standard deviation, *y *years, *mo *months

The functional scores including IKDC, Lysholm, VAS, HSS, and Tegner activity scale were summed up in Table 2. Most results were significantly different between pre-operation and post-operation. Mean pre-operation IKDC score ranged from 36.3 [18, 23] to 57.9 [10], while from 55.5 [19] to 92.6 [10] for post-operation. Moon et al. [19] didn’t come out with different results, because they just followed up for 2 years, which may be short to see the difference. Only Lee et al. [20] recorded a pre- and post-operative IKDC score in M and T group, but they did not make a statistical comparison. In M group, mean pre-operation Lysholm score ranged from 51.3 [19, 24] to 58.1 [18, 25], while from 72.0 [19] to 92.9 [10] for post-operation. Ke et al. [9] and Lee et al. [20] reported this score both pre- and post-operation, but neither compared directly. Only 2 [18, 19] studies in M group reported VAS scores, and 1 [18] of them got better after the operation. There was only 1 study in each group reporting HSS score. One [10] in M group significantly improved after the operation, while another [9] in M and T group didn’t. The Tegner activity scale was reported in 4 (36.4%) articles of M group and 1 (25.0%) article of M and T group. Most results [11, 12, 18] in M group were significant, while the one [20] in M and T group didn’t compare.

**Table 2:** Clinical outcome scores^a^

Author(s)	IKDC score (mean ± SD)		Lysholm score (mean ± SD)		VAS (mean ± SD)		HSS score (mean ± SD)		Tegner activity scale (mean ± SD)	
	Pre-operation	Post-operation	Pre-operation	Post-operation	Pre-operation	Post-operation	Pre-operation	Post-operation	Pre-operation	Post-operation
M group										
Kim, et al. [10]	**57.9 ± 3.3**	**92.6 ± 4.0**^b^	**54.9 ± 4.1**	**92.9 ± 3.9**	-	-	**55.0 ± 5.1**	**92.8 ± 3.0**	-	-
Lee, et al. [11]	**43.8 ± 7.6**	**78.1 ± 7.3**	**56.8 ± 7.6**	**86.5 ± 5.0**	-	-	-	-	**4.4 ± 1.0**	**4.8 ± 1.5**
Chung, et al. [12]	**40.1 ± 7.9**	**73.7 ± 11.1**	**52.3 ± 9.1**	**84.3 ± 12.1**	-	-	-	-	**2.7 ± 0.8**	**3.6 ± 1.1**
Chung, et al. [13]	**40.4 ± 7.0**	**74.2 ± 10.4**	**52.1 ± 8.2**	**84.5 ± 11.0**	-	-	-	-	-	-
Kim, et al. [14]	**39.7 ± 14.9**	**75.2 ± 18.8**	**51.7 ± 15.7**	**80.9 ± 15.8**	-	-	-	-	-	-
Chung, et al. [7]	**41.0 ± 9.6**	**63.7 ± 20.6**	**52.3 ± 10.9**	**77.1 ± 24**	-	-	-	-	-	-
Ulku, et al. [16]	-	-	56.1 ± 8.2	88.4 ± 4.1	-	-	-	-	-	-
Furumatsu, et al. [18]	**36.3 ± 16.0**	**64.7 ± 12.4**	**58.1 ± 9.6**	**86.4 ± 8.6**	**40.9 ± 20.5**	**11.2 ± 12.3**	-	-	**1.5 ± 1.0**	**3.0 ± 0.9**
Moon, et al. [19]	37.7 ± 15.8	55.5 ± 14.6	51.3 ± 24.9	72.0 ± 18.8	55.1 ± 24.5	15.5 ± 15.6	-	-	-	-
M and T group										
Ke, et al. [9]	-	-	36.3 ± 4.3	88.9 ± 4.5	-	-	38.5 ± 4.0	85.3 ± 3.4	-	-
Lee, et al. [20]	38.2 ± 16.4	80.5 ± 16.3	43.2 ± 14.0	87.1 ± 13.9	-	-	-	-	3.7 ± 1.1	5.1 ± 1.1

^a^*IKDC* International Knee Documentation Committee, *K-L* Kellgren-Lawrence, *VAS* visual analogue scale, *HSS* Hospital for Special Surgery, *SD *standard deviation

^b^The statistics with significant differences are represented in bold (*P* < 0.05)

**Fig. 2:**
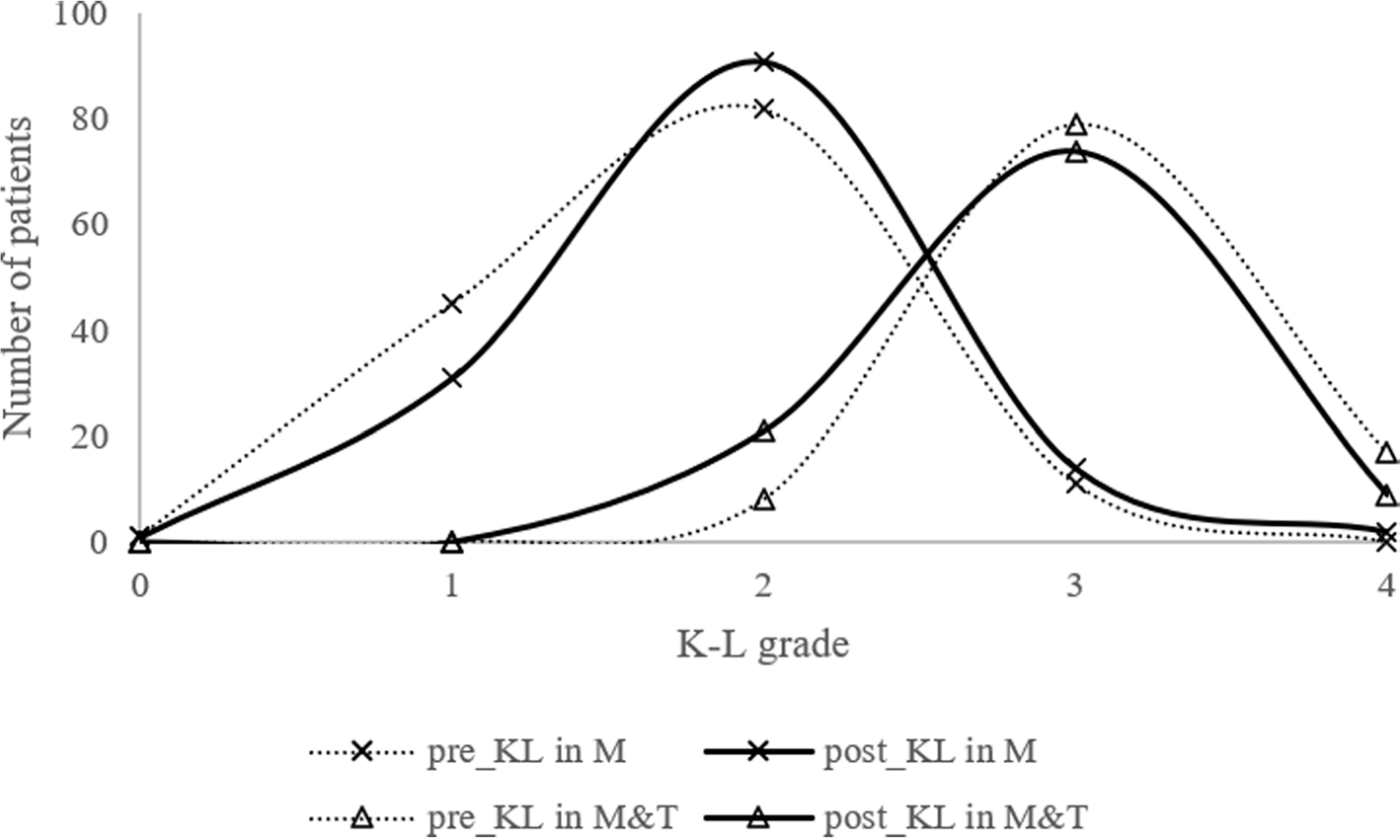
The change of K-L grade in two groups

The radiological outcomes composed of K-L grade, mechanical axis, medial joint space, meniscal extrusion, and healing status of medial meniscus were shown in Table 3. Four articles [12–14] had significant improvements in K-L grade after repair surgery, while others didn’t compare directly or had no significant difference. The distribution of patients with different K-L grades with about 2-year follow-up was shown in Fig. 2 [9–11, 17, 20, 21]. Neither group made significant progress although the M and T group had a larger mechanical axis before surgery than M group, which could attribute to the selection bias brought by indication of HTO. There was no obvious difference in medial joint space between the 2 groups, except Chung et al. [12] got significantly narrower results. The pre- and post-operation meniscal extrusions were mentioned in 3 [10, 15, 16] studies in M group and 2 [9, 21] in M and T group. In addition, 5 studies just reported pre- or post-operation data. The repair operation tended to decrease meniscal extrusion, but the sample was too small. The healing status of menisci could be seen in 6 articles, while Lee et al. [20] reported different classification methods from others.

**Table 3:** Radiologic outcomes^a^

Author(s)	K-L grade (0/1/2/3/4)		mechanical axis (mean ± SD)		medial joint space (mean ± SD)		meniscal extrusion (mean ± SD)		healing status (complete/partial/none)
	Pre-operation	Post-operation	Pre-operation	Post-operation	Pre-operation	Post-operation	Pre-operation	Post-operation	
M group									
Kim, et al. [10]	0/14/31/0/0	0/9/33/3/0	-	-	-	-	4.2 ± 0.9	2.2 ± 0.9	23/8/0
Lee, et al. [11]	0/18/27/5/0	0/10/37/3/0	2.5 ± 2.2	-	-	-	-	0.2 ± 1.1	23/25/2
Chung, et al. [12]	**6/25/6/0/0**	**0/11/20/6/0**^b^	3.6 ± 2.5	-	**4.8 ± 1.0**	**4.1 ± 1.1**	-	-	-
Chung, et al. [13]	**5/31/11/0/0**	**0/10/25/12/0**	-	-	4.7 ± 1.0	3.9 ± 1.0	-	4.3 ± 1.5	22/11/0
Kim, et al. [14]	**1/9/11/0/0**	**0/8/5/8/0**	3.2 ± 1.4	-	4.7 ± 1.3	4.1 ± 1.2	2.2 ± 1.5	-	-
Chung, et al. [7]	4/25/8/0/0	-	3.7 ± 2.3	-	4.8 ± 1.1	-	-	-	-
Hiranaka, et al. [15]	-	-	3.0 ± 1.7	-	-	-	3.8 ± 0.9	3.8 ± 1.1	-
Ulku, et al. [16]	-	-	-	-	-	-	**3.6 ± 0.5**	**2.4 ± 0.6**	-
Dzidzishvili, et al. [17]	1/13/24/6/0	1/12/21/8/2	-	-	-	-	-	-	-
Moon, et al. [19]	11/37/3/0/0	3/20/25/3/0	1.8 ± 1.9	0.6 ± 0.9	-	4.5 ± 1.4	3.5 ± 1.0	-	27/22/2
M and T group									
Ke, et al. [9]	0/0/8/20/2	0/0/14/16/0	3.3 ± 1.2	-3.9 ± 0.9	3.8 ± 1.1	3.7 ± 0.8	4.1 ± 1.5	4.0 ± 1.4	-
Lee, et al. [20]	0/0/0/43/6	0/0/2/42/5	-	-	2.3 ± 1.4	2.7 ± 1.5	-	-	-
Lee, et al. [21]	0/0/0/16/9	0/0/5/16/4	6.3 ± 2.2	1.9 ± 1.2	3.4 ± 1.0	3.7 ± 1.2	4.6 ± 1.9	4.5 ± 1.3	10/9/6
Suh, et al. [22]	-	-	6.8 ± 1.9	-1.3 ± 1.8	3.2 ± 1.4	3.5 ± 1.0	-	-	-

^a^*K-L* Kellgren-Lawrence, *MMPRT* medial meniscal posterior root tear, *HTO* high tibial osteotomy, *SD* standard deviation

^b^The statistics with significant differences are represented in bold (*P* < 0.05)

## Discussion

The findings of this systematic review suggest that the outcomes of MMPRT repair can be excellent, regardless of whether HTO is performed. Furthermore, the K-L grade progression at two-year follow-up was found to be comparable between the M and T and HTO groups, even though the pre-operative osteoarthritis in the M and T group was more severe.

From the perspective of clinical outcomes, there was no difference between MMPRT repair only and combination with HTO. Multiple reviews reported similar results with MMPRT repair only: Edwards et al. [26] reported an improvement of IKDC from 43.9 to 75.7 and Lysholm from 54.8 to 85.1 at a mean follow-up of 34 months; Chang et al. [27] reported that at midterm follow-up of 44 months, IKDC improved from 42.3 to 71.4, Lysholm from 53.4 to 84.1, HSS from 57.6 to 91.8, and Tegner activity scale from 2.8 to 3.8; and Krivicich et al. [28] reported a long-term follow-up of 64.8 months, with an IKDC score of 74.1. Kyun-Ho et al. [29] also compared the difference in HTO with or without MMPRT repair, finding no significant difference between groups in Lysholm and WOMAC, although the HTO with MMPRT repair group still had a higher mean Lysholm score. In our review, most articles in the MMPRT repair only (M) group had significantly improved Lysholm scores, while the improvement was not significant in the combination (M and T) group. However, the baseline Lysholm scores were worse in the M and T group, indicating that this difference was not an advantage of the M group.

The results of K-L grade supported the potential relief of progression of osteoarthritis through the repair of MMPRT. Moon et al. [30] documented that pullout repair of MMPRT improved clinical outcomes significantly. However, there were still 3 out of 31 patients having chondral lesions after the surgery, as well as meniscus extrusion progression being related to preoperative meniscus extrusion. Contrarily, Krych et al. [31] reported that 52 patients with MMPRT receiving non-operative therapy resulted in 31% of patients undergoing total knee arthroplasty (TKA) at a mean of 30 months after diagnosis, with K-L grade and arthritis becoming more severe with time, and 87% of patients failing in the end. Despite this, the benefit of surgical repair was still disputed. Masuda et al. [32] found that medial meniscus posterior extrusion increased when the knee flexed to 90 degrees in MMPRT, and Hopkins et al. [33] reported a high portion of patients having K-L progression after pullout repair. Additionally, it is unclear if the combination of MMPRT repair and HTO is more effective than either treatment alone. Kim et al. [34] concluded that HTO could yield similar results in both the intact meniscus group and the MMPRT group. Thus, more convincing studies are required to elucidate the functions of MMPRT repair and HTO.

The potential advantage of HTO was to correct the lower limb mechanical axis, which was essential for normal biomechanical functions. Moon et al. [30] confirmed that patients with varus alignment of > 5° had poorer results than those with varus alignment of < 5°. Theoretically, HTO could correct the malignment of the lower limb, thus improving the stress distribution on the meniscus and accelerating its healing. Chung et al. [35] observed 37 MMPRT patients who underwent pullout repair for more than 10 years, 8 of whom underwent TKA. Compared to the others, these 8 had greater varus alignment degrees, larger portions, and more progression of meniscus extrusion values. They suggested that 5 degrees of varus and 0.7 mm differences in meniscus extrusion values between preoperative and 1-year postoperative values could be used as the cutoff values to predict failure of MMPRT repair. However, the clinical outcomes in more recent studies challenge this hypothesis. Ridley et al. [36] compared the outcomes of MMPRT patients, divided by whether concomitant HTO was performed and varus was greater than 5 degrees. They found that patients with HTO had worse outcomes, regardless of alignment contrary to the hypothesis. This suggests that the effect of HTO is unclear and the preoperative varus degree plays an important role, with 5 degrees not being a reliable predictor. In our results, patients’ K-L grades in M and T group were mainly concentrated on 3. A multicenter cohort study revealed that the K-L 2 grade and 3 grade had totally different cartilage morphologies [37]. Thus, we suggest taking K-L grade into account when distinguishing high-risk patients and deciding whether or not to do HTO, which has been taken into account in some articles [20].

Noticeably, the potential innovation of combining MMPRT with combined tibial surgeries might provide further benefits for patients with mechanical malalignment. Chiba et al. reported that tibial condylar valgus osteotomy (TCVO) can improve pain and activities of daily living, along with valgus correction of the lower extremity and stabilization of the femorotibial joint in advanced medial knee osteoarthritis [38]. It’s indications, detailed surgical techniques, and outcomes were also reported by Capella et al. [39]. This opens up the possibility of further exploring the clinical efficacy of combining TCVO with MMPRT and assessing if similar outcomes to those found in this study can be replicated in those patients.

This article had several limitations. Firstly, the heterogeneity of study procedures resulted in outcomes that could not be directly aggregated, making the analysis complex. Secondly, there was a lack of long-term studies, with most studies (83.3%) having a follow-up period of less than 5 years, preventing us from making conclusions about long-term prognosis. Thirdly, the majority of studies were retrospective and non-randomized comparative studies, introducing selection bias into the conclusions. Nevertheless, this article was the first to examine the effect of HTO on the results of MMPRT repair in terms of lower limb alignment. Although there were several limitations, this article firstly reviewed and compared the outcomes of MMPRT repair with and without HTO, which provided evidence for clinical decision-making. To draw more reliable conclusions, higher evidence studies such as randomized control trials and prospective cohort studies are needed in the future.

## Conclusion

The use of HTO as a supplement in treating MMPRT patients with severe osteoarthritis has been found to yield similar clinical and radiological outcomes to MMPRT repair alone. Nevertheless, it is still controversial as to which of the two treatments is better for patients’ prognosis. It is suggested to take K-L grade into account when choosing the most suitable treatment. To make more informed clinical decisions, large-scale randomized control studies should be conducted in the future.

## Data Availability

All data generated or analyzed during this study are included in this manuscript.

## Abbreviations

MMPRT: medial meniscal posterior root tear
HTO: High tibial osteotomy
NOS: Newcastle-Ottawa Scale
SD: Standard deviation
K-L: Kellgren-Lawrence
IKDC: International Knee Documentation Committee
K-L: Kellgren-Lawrence
VAS: Visual analogue scale
HSS: Hospital for Special Surgery
